# Fifty years of hemodialysis in Ghana—current status, utilization and cost of dialysis services

**DOI:** 10.1186/s12913-023-10154-x

**Published:** 2023-10-27

**Authors:** Elliot Koranteng Tannor, Kojo Hutton-Mensah, Priscilla Opare-Addo, Martin Kofi Agyei, Kwadwo Faka Gyan, Abdul-Jalil Inusah, Beatrice Irene Nyann, Kwabena Amo-Antwi, Valerie Luyckx, Ikechi Okpechi

**Affiliations:** 1 Department of Medicine, School of Medicine and Dentistry, Kwame Nkrumah University of Science and Technology, Kumasi, Ghana; 2 Renal Unit, Directorate of Medicine, Komfo Anokye Teaching Hospital, Kumasi, Ghana; 3 Department of Medicine and Therapeutics, Korle Bu Teaching Hospital, Accra, Ghana; 4 Department of Paediatric, University of Ghana Medical Centre, Accra, Ghana; 5 Department of Obstetrics and Gynaecology, Kwame Nkrumah University of Science and Technology, Kumasi, Ghana; 6 Renal Division, Brigham and Women’s Hospital, Harvard Medical School, Boston, MA USA; 7 Department of Paediatrics and Child Health, University of Cape Town, Cape Town, South Africa; 8 Department of Public and Global Health, Epidemiology, Biostatistics and Preventive Institute, University of Zurich, Zürich, Switzerland; 9 Division of Nephrology and Hypertension, Groote Schuur Hospital and University of Cape Town, Cape Town, South Africa; 10 Department of Medicine, Division of Nephrology and Immunology, University of Alberta, Edmonton, Canada

**Keywords:** Haemodialysis, Cost of dialysis, Regional distribution, Ghana

## Abstract

**Background:**

Kidney failure is common in Ghana. Haemodialysis (HD) is the most common treatment modality for survival. Although, HD has been available in Ghana for 50 years, the majority of patients who develop kidney failure cannot access it. We describe the state of HD, dialysis prevalence, its utilization and cost of HD after fifty years of dialysis initiation in Ghana.

**Methods:**

A situational assessment of HDs centres in Ghana was conducted by surveying nephrologists, doctors, nurses and other health care professionals in HD centres from August to October 2022. We assessed the density of HD centres, number of HD machines, prevalence of nephrologists, number of patients receiving HD treatment and the cost of dialysis in private and government facilities in Ghana.

**Results:**

There are 51 HD centres located in 9 of the 16 regions of Ghana. Of these, only 40 centres are functioning, as 11 had shut down or are yet to operate. Of the functioning centres most (*n* = 26, 65%) are in the Greater Accra region serving 17.7% of the population and 7(17.5%) in the Ashanti region serving 17.5% of the population in Ghana. The rest of the seven regions have one centre each. The private sector has twice as many HD centers (*n* = 27, 67.5%) as the public sector (*n* = 13,32.5%). There are 299 HD machines yielding 9.7 HD machines per million population (pmp) with a median of 6 (IQR 4–10) machines per centre. Ghana has 0.44 nephrologists pmp. Currently, 1195 patients receive HD, giving a prevalence of 38.8 patients pmp with 609(50.9%) in the private sector. The mean cost of HD session is US $53.9 ± 8.8 in Ghana.

**Conclusion:**

There are gross inequities in the regional distribution of HD centres in Ghana, with a low HD prevalence and nephrology workforce despite a high burden of CKD. The cost of haemodialysis remains prohibitive and mainly paid out-of-pocket limiting its utilization.

**Supplementary Information:**

The online version contains supplementary material available at 10.1186/s12913-023-10154-x.

## Background

Globally, the incidence and prevalence of chronic kidney disease (CKD) are on the increase [1]. The prevalence of CKD in Africa and sub-Saharan Africa are 15.8% and 13.9% respectively [2, 3]. In Ghana, the prevalence of CKD is 13.3%, with chronic glomerulonephritis, diabetes mellitus and hypertension as major causes [4–6]. When CKD progresses to kidney failure (KF), kidney replacement therapy (KRT) is required for survival and to improve quality of life [7, 8]. Kidney transplantation is the best modality of KRT as it improves survival and quality of life compared dialysis [9–11].

Globally, patients in Africa have the lowest access to KRT, with the lowest rates found in Central and Eastern Africa with1-3% of those requiring receiving it [12]. According to the Global Kidney Health Atlas (GKHA), only 4–10% of patients with KF can access KRT in low- and lower middle income countries(LMIC) [13]. Limited access to KRT results from unavailability and high cost in most parts of Africa, including Ghana [14]. Ghana does not have a sustainable kidney transplant program and haemodialysis (HD) is the most common modality for KF management [15, 16]. According to the first Ghana renal registry in 2017, 96.2% were on HD, 0.3% on PD and 3.5% had kidney transplant [15]. There is also inequitable distribution of HD services in Ghana [17]. HD is not reimbursed by the National Health Insurance Scheme (NHIS) [16].

Dialysis is also required in severe cases of acute kidney injury (AKI). AKI accounts for 5.05–24.9% of patients with kidney disease in Ghana [18, 19]. In a single centre study, HD was required in 40.5% of patients with kidney disease on admission, but only 14.5% could afford HD leading to in-hospital mortality of 45.6% [20]. HD requirement for AKI is usually short-term but the cost remains prohibitive with high rates of preventive deaths.

South Africa was the first country to start HD in Africa in 1957, followed by Egypt a year later [21] for AKI management. Countries like Kenya(1961), Nigeria (1965), Sudan (1968) and Libya (1972) then followed [22, 23]. Ghana initiated dialysis in 1972 [22] and by 2015, HD was available in three teaching hospitals and three private facilities [16]. By December 2016 there were 15 dialysis centres with 103 HD machines in the country [17]. However, the rates of transplantation and peritoneal dialysis have however not increased significantly [15].

In this study, we evaluate the current status of HD services in Ghana by regional distribution of services, prevalence of HD patients, cost of HD treatment, utilization of dialysis services and availability of nephrology workforce to deliver HD treatment across centres in Ghana after five decades of HD initiation.

## Methods

A cross-sectional situational survey was conducted across all HD centres in Ghana. Questionnaires were circulated and telephone calls were made between August-October 2022. The questionnaire was developed for the survey to understand the current state of dialysis in Ghana (Supplementary file 1). We sought data from nephrologists, doctors, nurses, and health care workers as key informants providing HD care in HD centres in Ghana, to ascertain the number of HD centres, number of HD machines in both public and private facilities, the presence of a resident/locum nephrologist, number of patients on HD and the cost per session of HD. The number of HD units that were previously operational but currently non-functional as well as new HD centres yet to start operation were also documented. The cost of HD was converted to United States dollars (US$) equivalent at the time of study in 2022 using an available currency converter [24]. Data were expressed as proportions, percentages, means ± standard deviation and medians and interquartile ranges as appropriate and analysis done with STATA version 16 [25]. Trends in the data with reference to previous studies conducted in Ghana [16, 17] were analyzed descriptively. Being a survey where no patient or personal data was collected, ethical clearance was waived by the institutional review board.

### Ghana’s demography and economy

Ghana is a country located in West Africa bounded by Burkina Faso (north), Togo (east), Côte d'Ivoire (west) and the Gulf of Guinea (south). Ghana has a surface area of 238,533km^2^, 16 regions and a population of 30.8 million people according to the 2021 Population and Housing Census [26]. Greater Accra is the smallest region measuring 3,245 km^2^ and hosts the national capital Accra with a population of 5,455,692 [27]. The Savannah region is the largest with a surface area of 35,862 km^2^ and located in the middle belt of Ghana, with a population of 658,946 people. The Ashanti region is also in the middle belt with a population of 5440,463 and a surface area of 24,869 km^2^ [27].

According to the World Bank, Ghana has a gross domestic product (GDP) of US$77.59 billion and a GDP per capita of US$2,445.39 in 2021 [28]. Hence Ghana is categorized as a LMIC. The daily minimum wage at the time of the study was GHS13.53(US$2.06) per day or GHS365.31(US$55.7) per month [29].

Ghana spent 7.6% of its budget and 1.38% of its GDP on health in 2022 [30] which is less than the recommended 15% by the Abuja declaration [31]. Healthcare is financed largely by progressive taxes for 50% of health care funding, however with out-of-pocket payment accounting for about 45% of health services funding in Ghana [32]. The national health insurance scheme does not reimburse for HD care [16, 17] leading to low utilization of HD services in Ghana.

## Results

There are 51 HD centres in Ghana including 37(72.5%) private centres and 14(27.5%) public centres. The majority of the HD centres are in the Greater Accra region 33(64.7%) with 9(17.7%) in the Ashanti region of Ghana. Only 40(78.4%) centres are currently functional, 9(17.7%) centres are not operational and 2(3.9%) new centres have been set up but not yet operational at the time of the survey (Table 1). Haemodialysis services offered in Ghana is mainly intermittent in-centre haemodialysis.

**Table 1:** The distribution of haemodialysis centres, patient prevalence, nephrologists and cost of dialysis in Ghana

Variable	Measure
Haemodialysis machines in Ghana (n)	299
Haemodialysis machines—(pmp)	9.7
Private centres [n (%)]	27 (67.5)
State owned centres [n (%)]	13 (32.5)
Haemodialysis centres without nephrologist [n (%)]	23 (57.5)
Prevalence of nephrologists in Ghana (pmp)	0.44
Haemodialysis machines in Ghana	6 (4–10)^a^
Haemodialysis machines in private centres	6 (4–10)^a^
Haemodialysis centres in state-owned sector	8 (3–10)^a^
Patients per haemodialysis centres	20 (11.5–36.5)^a^
Patients per haemodialysis centres in private centres	14 (10–25)^a^
Patients per haemodialysis centres in state-owned centres	25 (14–53)^a^
Mean cost of haemodialysis session in Ghana	53.9 ± 8.8^b^
Mean cost of haemodialysis in private centres	56.7 ± 7.9^b^
Cost of haemodialysis in state-owned centres	48.2 ± 7.9^b^

*Abbreviations*: *n* number, *IQR* interquartile range, *US $* United States Dollars, *SD* standard deviation, *per* million population, *pmp* [Median; (IQR)]^a^, (mean ± SD)^b^

**Fig. 1:**
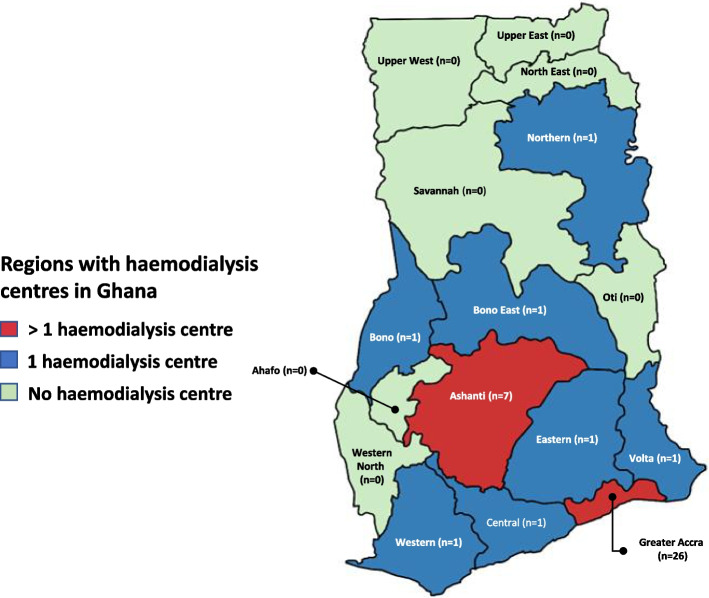
The distribution of functional haemodialysis centres by region in Ghana in 2022. Created with mapchart.net

Of the 40 functional centres in Ghana, the majority are in the Greater Accra region and Ashanti regions with 26(65%) and 7(17.5%) respectively. The seven other regions have one HD centre each (Fig. 1).

**Fig. 2:**
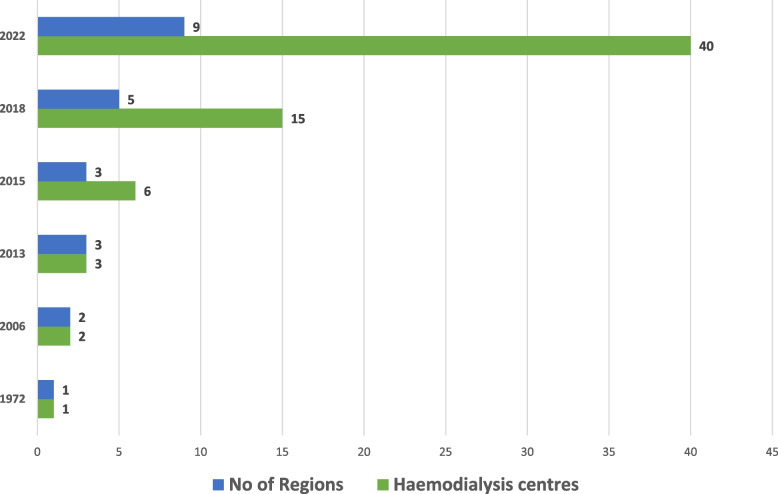
Growth of the number of hemodialysis centers across regions in Ghana from 1972

There has been a steady increase in regional distribution of HD centres from one centre in the greater Accra region in 1972 to centers being present in 9 out of the 16 regions in 2022 (Fig. 2).

**Fig. 3:**
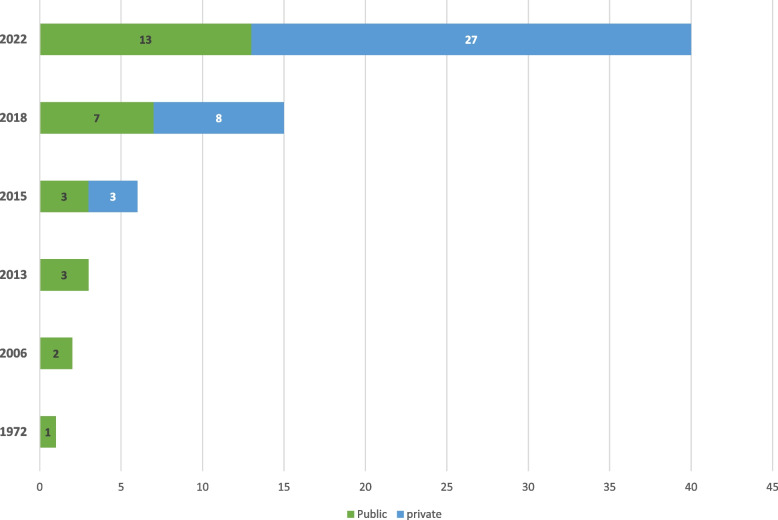
Trend in haemodialysis centres for private and state-owned centres from 1972 in Ghana

Private facilities were introduced in 2015 and have been increasing much faster than public facilities which were introduced in 1972 (Fig. 3).

There are 0.17 HD centres per 1000km^2^ in Ghana (assuming equitable distribution). Greater Accra again has the highest density of HD centres (8.01 per 1000km^2^) followed by the Ashanti region (0.29 per 1000km^2^). Bono East and Northern regions have the lowest density of centres (0.04 HD centres per 1000km^2^ each). The seven regions without HD centres make up 5,702,791(18.5%) of the population and cover 98,587km^2 ^(41.3%) surface area of Ghana (Table 2).

**Table 2:** Regional distribution of functional dialysis centres and patients per million population in Ghana

Region	Population (%)	Population (n)	Surface area km^2^	No. of HD centres	No of HD machines	No. of patients	Patients prevalence (pmp)	Dialysis machines (pmp)	Dialysis centres per 1000km^2^
Greater Accra	17.69	5,455,692	3,245	26	227	875	160.4	50.77	8.01
Ashanti	17.64	5,440,463	24,389	7	39	96	17.6	7.17	0.29
Bono East	3.92	1,203,400	23,257	1	3	12	9.97	2.49	0.04
Bono	3.90	1,208,649	11,107	1	2	4	3.3	1.65	0.09
Central	9.28	2,859,821	9,826	1	10	53	18.5	3.50	0.10
Eastern	9.49	2,925,653	19,323	1	8	25	8.5	2.73	0.05
Northern	7.50	2,310,939	25,448	1	5	90	3.9	2.16	0.04
Volta	5.38	1,659,040	9,504	1	3	20	12.1	1.81	0.11
Western	6.68	2,060,585	13,847	1	2	20	9.7	0.97	0.07
Upper East	4.22	1,301,226	8,842	0	0	0	0	0	0
Upper west	2.92	901,502	18,476	0	0	0	0	0	0
Western North	2.86	880,921	10,074	0	0	0	0	0	0
Oti	2.42	747,248	11,066	0	0	0	0	0	0
North-East	2.14	658,946	9,074	0	0	0	0	0	0
Savanna	2.12	653,266	35,862	0	0	0	0	0	0
Ahafo	1.83	554,668	5,193	0	0	0	0	0	0
Regions without dialysis	18.52	5,702,791	98,587	0	0	0	0	0	0
**Total**	**100**	**30,792,608**	**238,533**	**40**	**299**	**1195**	**38.8**	**9.7**	**0.17**

*Abbreviations*: *HD* haemodialysis, *pmp* per million population, *km* kilometers

There are 299 HD machines with 6(IQR 4–10) machines per centre, yielding 9.7 HD machines per million population(pmp) in Ghana (Table 1).

**Fig. 4:**
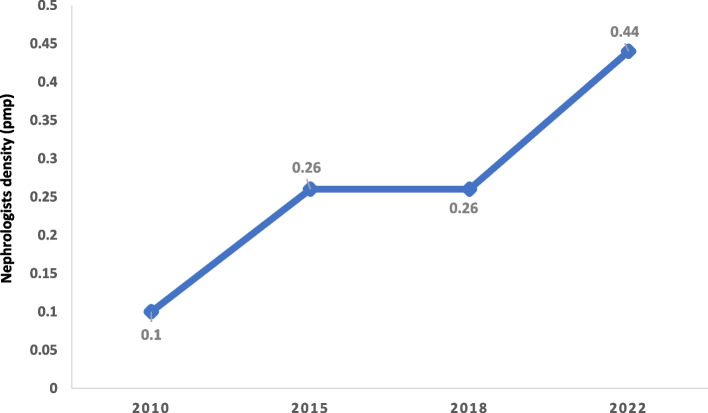
Prevalence of nephrologists per million population in Ghana from 2010—2022

Regionally, Greater Accra has the highest number of HD machines pmp with 50.8pmp followed by Ashanti region with 7.2 HD machines pmp. The Western region has the lowest number of HD machines with 0.97pmp. Ghana has 15 nephrologists for a population of 30.8million with a prevalence of 0.44 nephrologists pmp (Table 1). Over half 23(57.5%) of the functional dialysis centres do not have a resident or locum nephrologist associated with the centre. The prevalence of nephrologists pmp has increased marginally since 2010 (Fig. 4).

There are 1195 patients (private—609; public—586) receiving HD in Ghana. The HD prevalence is 38.8pmp in Ghana. The Greater Accra region has the highest prevalence of patients receiving HD (160.4pmp), followed by the Ashanti region (17.6 pmp). The least prevalent region is Bono East with a patient prevalence of 3.3pmp. There are no dialysis services for seven regions with a population of 5.7million people (Table 2). The mean cost of HD session in Ghana is US $53.9 ± 8.8. The cost in public centres is US$48.2 ± 7.9 and US$56.7 ± 7.9 in private centres.

## Discussion

After 50 years of HD initiation in Ghana, this study assessed the regional distribution, status of HD services, patient prevalence on HD, nephrology workforce, and cost of HD in Ghana. Since 2016, the number of functional HD centres have increased from 15 to 40 and HD machines have increased from 103 to 299 in 2022 [17]. Gross inequities persist in the distribution of HD centres with very low nephrology workforce and high cost of HD.

The HD machines pmp have increased from 4.2pmp in 2016 [17] to 9.7pmp in 2022. The increase in HD centres has been largely contributed by the private sector expansion by 237.5% as compared to 85.7% increase in the public sector. Though patients are almost equally distributed in private and public centres, the median number of patients are higher in public centres compared to private centres. This likely reflects the higher cost of HD in the private centres. Seven regions occupying 41.3% of Ghana’s surface area and inhabited by 18.5% of the population have no HD centres [27]. Patients in these areas have to travel very long distances to access HD adding on to the cost of care lower utilization leading to poor quality of life, morbidity and mortality [7, 33].

Geographically, there is inequitable distribution of HD centres in Ghana. Countrywide, there are 17 HD centres per 100,000km^2^ while Greater Accra has 8 HD centres per 1000km^2^. This inequity is driven by population density and the ability to pay for services, hence HD is not accessible or affordable for the majority. Accessibility, availability, affordability, acceptability and accommodation make up the Penchansky’s conceptual model required for health service utilization [34]. Access refers to all the factors between the perception of need for the HD service to the utilization of the service [35].

The HD patient prevalence in Ghana is 38.8pmp, a marginal increase from 23.6pmp reported in 2017 [15]. This remains lower than the average prevalence in Africa of 79pmp and a global average of 296 in a systematic review [12]. Ghana’s prevalence is lower than the average for LMIC(68pmp). Ghana is comparable with some countries in sub-Saharan Africa and low income settings with a prevalence of less than 20pmp [36].

From global ballpark figures, it may be estimated that around 0.05% of the population in LMICs would develop KF [12]. With a population of 30.8 million people, Ghana has about 15,400 patients with KF. The 1,195 patients on HD in our survey represents only 7.8% of estimated patients with KF requiring HD in Ghana. Hence, some 92% of patients with KF have no access to KRT, consistent with the 90% reported for LMIC by GKHA [13] as PD and kidney transplants are in the minority [15].

In Ghana, the monthly minimum wage (US$55.7) can pay for only a session of HD (US$53.9) [29]. The cost of dialysis imposes substantial household burden on HD patients [37]. In a single centre study in Ghana, only 14.5% were able to utilize HD [20] resulting in high mortality of 32.9–45.6% [20, 33]. Due to high cost, most patient are unable to afford the prescribed three-times a week haemodialysis and settle for twice-a-week or even once-a-week haemodialysis in most cases.

The cost of HD excludes other direct medical costs which can be categorized as (i) medications such as oral haematinics, intravenous iron, subcutaneous erythropoietin, anti-hypertensives, medications for the management of mineral bone disease and (ii) vascular access-related such as arteriovenous fistula, arteriovenous graft, HD tunneled and temporary catheter costs. The average annual cost of HD for the required three sessions per week in Ghana is US$8,408.4 and US$5,605.6 for two sessions/week per person. The cost of HD services can be grouped into four categories. These are direct medical, direct non-medical, indirect and intangible costs [38, 39]. For HD treatment, the direct medical costs include staff, dialyzers, dialysis tubing, nephrologist consultation, capital cost for HD machines, cost of hospitalization and associated laboratory investigations, medications and vascular access. Direct non-medical costs include building costs, facility utilities such as electricity, water, administrative and overhead costs. Indirect costs are related to loss of productivity by the patients and caregivers as a result of the disease, transport cost and relocation. Intangible cost is related to the pain, symptoms and quality of life [37].

The cost of HD in Ghana is higher than in India (annual cost of US $3,423.79) and lower than in Nigeria (US $42,784.91) and Kenya (US$16,845.10) [40]. The cost per HD session is also lower than in Tunisia (US$65) and South Africa (US$140) [41]. In Ghana, the cost of HD is borne directly by most patients out-of-pocket as the NHIS does not reimburse for HD. Some private insurance and philanthropists support some patients with KF in some HD centres in Ghana [42]. NHIS reimburses admission cost, some medications and laboratory tests [16].

Increasingly, countries in sub-Saharan Africa even with lower GDP per capita than Ghana (US$2,445.5) such as Kenya (US$2,006.8) and Tanzania (US$1,135.5) [43, 44] have initiated universal coverage with twice-a-week dialysis services. This has led to a dramatic increase in dialysis utilization and prevalence in these countries.

Countries in sub-Saharan Africa adopt various approaches to provide HD services for patients with KF. This includes universal coverage of dialysis for AKI and KF (Kenya, Sudan, Malawi), government subsidy for HD (Senegal, Ethiopia, Cameroun), universal coverage for AKI only (South Africa, Ethiopia) and state coverage for chronic HD under limited conditions (South Africa), while others like Ghana, Nigeria, Burundi and Democratic republic of Congo provide no state coverage [45].

With the desired political will, Ghana could support the management of patients with KF far more than in its current state. If the expected 15,400 patients with KF are treated with twice-a-week HD at US $53 per session, about 100 sessions will be required per person per year. It will cost US $81,620,000 per year (US $5,300 per patient) which is 7.4% of the health budget. This might overburden the health budget considering other disease conditions of similar importance, hence governments may consider about 2% or more of the health budget (US $22 million) which can manage about 4,150 patients with KF per year initial rationing of dialysis like in South Africa [46] bearing in mind its challenges. The cost per unit of HD can also substantially be reduced if the import duties were reduced, and with large volume purchases decreasing further the cost per session. Pricing should also be further negotiated with dialysis companies. Local production of dialysis supplies should be explored to improve dialysis service utilization.

In 2022, US$1.1 billion was allocated to health in Ghana which is 7.6% of budget spending and 1.38% of GDP [30]. This is less than the 15% recommended by the Abuja declaration [31]. There is the need to increase the overall budgetary allocation to health as the cost of KRT for all who require it would have significant opportunity costs for other diseases [45].

Despite the limited availability of HD services, some existing HD facilities are folding up due to multiple reasons including unavailability or high cost of HD consumables due to high import duties and weak local currency, patients’ inability to pay realistic prices per session for HD, high property cost, administrative and labour costs making it difficult to break even to remain viable as well as the shortage of trained personnel such as dialysis nurses, technicians and nephrologists. The majority of HD centres do not have an attending nephrologist. Though the nephrologist prevalence has increased to 0.44 pmp in 2022 from 0.26 pmp in 2016 [17], it is still lower than the average of 0.5pmp in sub-Saharan Africa [13]. Ghana like most countries in sub-Saharan Africa needs more nephrologists to improve kidney care [47]. The nephrologists pmp in Ghana, a LMIC is currently only higher than in LIC (0.2pmp) but lower than in average LMIC (1.6pmp), UMIC (10.8pmp) and HIC (23.2 pmp) [13]. To improve the care and management of kidney disease, and to achieve kidney health for all in Africa, efforts should be put into investment in kidney health care, training of more nephrologists, task shifting in areas with less nephrology workforce, improving access to KRT all across the country and most importantly improving community engagement with education on disease prevention [47, 48]. The International Society of Nephrology (ISN) has improved the nephrology workforce in sub-Saharan Africa through the ISN fellowship program with positive impact [49] but there is still the need for more nephrology workforce in Ghana and most parts of sub-Saharan Africa [50].

In sub-Saharan Africa, efforts should be channeled towards sustainable preventive programs, health education and community engagement [48] to improve early detection and improve kidney health for all with optimal support for those who develop kidney failure [47].

This study has multiple strengths and few limitations. A major strength is that this situational survey results represents the most up-to-date source of information. Information represents a cross-sectional snapshot at the time of the survey which may change with time. It also possible that there may be a few patients who were on acute dialysis in some centres during the survey. Estimates of the locations of these dialysis centres per 1000 km square were based on the assumptions of equal distribution of the present dialysis centres. However, these fluctuations and estimations are unlikely to fundamentally change the overall message of poor access to HD and geographic inequities. This study therefore provides essential data for advocacy to improve kidney care in Ghana and sub-Saharan Africa.

### Recommendation

There is the need for more state support for patients with KF in Ghana. This could include complete funding of some patients with KF, subsidization of cost of HD consumables, strengthening the nephrology workforce, improving renal transplantation programs, establishment of dialysis centres in regions without any and supporting defunct HD centres to restart operations. Ghana should adopt strategies to support HD services through the NHIS as practiced in Kenya, Tanzania and South Africa [43–45]. Until then, efforts should be channeled towards prevention of kidney disease through public health approaches [48]. Universal health coverage for all is critical for early identification of risk factors and the provision of equitable access to early interventions to delay the progression of kidney diseases. There is the need for good governance, multiple stake-holder engagements, transparency and a universal commitment to improve the right to kidney care in Ghana [51].

## Conclusion

There have been advances in kidney care in Ghana for the past 50 years but gross inequity still exists. The HD prevalence in Ghana is below the average for LMICs and Africa. Cost of HD is still prohibitive but with more government support kidney care in Ghana and sub-Saharan Africa will substantially improve.

### Supplementary Information

**Additional file 1.** Survey questionnaire on the state of haemodialysis in Ghana.

## Data Availability

All data generated or analyzed during this study are included in this published article.

## Abbreviations

HD: Haemodialysis
PMP: Per million population
USD: United States Dollars
CKD: Chronic kidney disease
KF: Kidney failure
KRT: Kidney replacement therapy
GKHA: Global Kidney Health Atlas
LMIC: Low and lower middle income countries
AKI: Acute kidney injury
GDP: Gross domestic product
NHIS: National health insurance scheme
ISN: International Society of Nephrology
