# Supplementary material for: Fifty years of hemodialysis in Ghana—current status, utilization and cost of dialysis services
Source: BMC Health Serv Res. 2023 Oct 27;23:1170. doi: 10.1186/s12913-023-10154-x (PMC10612280; doi:10.1186/s12913-023-10154-x)
Supplement: Supplementary file 1 — Additional file 1. Survey questionnaire on the state of haemodialysis in Ghana. [file 12913_2023_10154_MOESM1_ESM.pdf]

Supplementary File 1

Study title - Fifty years of hemodialysis in Ghana - current status, utilization and cost of dialysis services

Survey questionnaire

1. Name of facility

   …………………………………………………………………………………………...

2. Personnel in-charge of the unit

   ………………………………………………………………………………………..

3. Are you a private or public facility? Private [ ] Public [ ]
4. In which city is the facility located………………………………………………………
5. In which region is the facility located? ………………………………………...........
6. Is the centre currently functional or operational? Yes [ ] No [ ]
7. If not, why are you not currently in operation reason?..

   …………………………………………………………………………………………

8. Do you have an attending nephrologist? Yes [ ] No [ ]
9. How many dialysis machines are currently functional in your facility? ……………..
10. How many patients are currently on dialysis in your facility? ……………….........
11. Cost of session of haemodialysis (GHS)? ……………………………………….....

Thank you very much for your time!
